# Impaired Recognition of Metrical and Syntactic Boundaries in Children with Developmental Language Disorders

**DOI:** 10.3390/brainsci9020033

**Published:** 2019-02-05

**Authors:** Susan Richards, Usha Goswami

**Affiliations:** Centre for Neuroscience in Education, University of Cambridge, Cambridge CB2 3EB, UK; ucg10@cam.ac.uk (U.G.)

**Keywords:** language disorder, rhythm, prosody

## Abstract

In oral language, syntactic structure is cued in part by phrasal metrical hierarchies of acoustic stress patterns. For example, many children’s texts use prosodic phrasing comprising tightly integrated hierarchies of metre and syntax to highlight the phonological and syntactic structure of language. Children with developmental language disorders (DLDs) are relatively insensitive to acoustic stress. Here, we disrupted the coincidence of metrical and syntactic boundaries as cued by stress patterns in children’s texts so that metrical and/or syntactic phrasing conflicted. We tested three groups of children: children with DLD, age-matched typically developing controls (AMC) and younger language-matched controls (YLC). Children with DLDs and younger, language-matched controls were poor at spotting both metrical and syntactic disruptions. The data are interpreted within a prosodic phrasing hypothesis of DLD based on impaired acoustic processing of speech rhythm.

## 1. Introduction

Children with Developmental Language Disorder (DLD) have persistent difficulties with learning language that are not associated with a known condition, such as sensori-neural hearing loss or Autism Spectrum Disorder [1]. Prevalence of the disorder is estimated at approximately 7% in primary school populations [2,3,4], and children with DLD can face a variety of challenges in accessing education and employment. Children with DLD typically have difficulty with the accurate processing and production of grammatical structures in speech [5,6,7].

Although the implications of having DLD are well-documented, and DLD is found across languages, the underlying causes are as yet unclear. A range of perceptual and cognitive hypotheses have been proposed, including impaired rapid auditory processing [8], impaired phonological memory [9] and genetically determined grammatical deficits [7]. One aspect of language processing that has not attracted significant research attention is the processing of language rhythm. The concept of language rhythm is not consistently defined in the literature and has often been regarded as a purely temporal phenomenon [10]. Others, however, have conceptualised linguistic rhythm in terms of the patterning of syllable prominence, with some syllables being acoustically more prominent than others [11]. Regarding the rhythm of spoken English, syllable prominence can be thought of in terms of strong or stressed syllables (the more prominent) and weak or unstressed syllables (the less prominent). For example, in the word baNAna, the second syllable ‘NA’ is more prominent than the first syllable ‘ba’ and third syllable‘na’. Accordingly, this word has a weak-strong-weak rhythmic structure, with the second syllable ‘NA’ carrying the primary stress. The patterning of strong and weak syllables across words, phrases and sentences thus contributes to the perception of language rhythm in addition to temporal factors. These patterns, made up of strong and weak syllables, can be grouped hierarchically into larger units via prosodic feet, in which one or more weak syllables is grouped with a strong syllable to form a temporal unit. This concept is a familiar one in certain kinds of poetry, in which patterns of recurring syllable rhythms are grouped to fit a higher-order temporal structure, for example via trochees (strong-weak syllable groupings repeating) or dactyls (strong-weak-weak groupings repeating). The pattern of groupings of strong and weak syllables into temporal units is commonly referred to as ‘metre’. For the English nursery rhyme ‘Jack and Jill went up the hill’, a perfect metrical poem, a trochaic rhythm is used, whereas for the nursery rhyme ‘Pussycat pussycat where have you been?’ a dactyl structure is repeated.

There are sound theoretical reasons for regarding efficient rhythmic processing as a key foundation skill for language development, making the study of the potential impacts of an early difficulty with efficient rhythmic processing of interest in attempting to understand developmental language disorders. Infants are exposed to the rhythmic aspects of language before birth and as newborns are able to use rhythmic properties to differentiate between languages [12,13]. Rhythmic sensitivity is accordingly considered a precursor of language acquisition, with the earliest representations of the speech signal encoding its rhythmic structure. Subsequent aspects of language, such as semantics and syntax, may be scaffolded onto these rhythmic representations [14]. Infants have been shown to use rhythmic aspects of language to establish structured linguistic representations at the level of word boundaries [15], lexical representations [16,17] and larger-grained grammatical units, such as phrases and clauses [18,19]. If rhythm is able to act in a bootstrapping role for subsequent language, then a difficulty in processing rhythm at the earliest stages of development could have a significant impact on the child’s subsequent trajectory of language development. In this study, we investigate the potential impact that a rhythmic processing difficulty might have at the interface of rhythm and syntactic structure. This is of particular interest since children with DLD typically present with difficulties in the accurate processing and production of linguistic syntax [20], and are known to have difficulties in processing linguistic stress patterns [21]. Accordingly, it is possible that difficulties in processing prosodic phrasing and prosodic hierarchies dependent on stress patterning may underlie their syntactic difficulties [22].

Impaired auditory sensory processing skills in children with DLD appear to contribute to their impaired processing of syllable stress patterns [21]. Four key acoustic parameters contribute to the perception of stress: frequency, intensity, duration and amplitude envelope rise time (AERT) [23]. Stressed syllables tend to be of a higher frequency than unstressed syllables, have longer durations and are of a higher intensity than unstressed syllables [23]. The fourth parameter, AERT, refers to the length of time between the between the onset of a sound and the point at which its amplitude reaches peak intensity. In speech, this is typically measured as the rise in amplitude from the beginning of a syllable until the speaker reaches the peak of the syllable nucleus (vowel). Stressed syllables have larger rise times, with a greater change in amplitude until the amplitude peaks at the syllable nucleus, whilst unstressed syllables have smaller changes in amplitude before the peak of the nucleus is reached. In order to speak deliberately to a rhythm, the speaker times their production of the rise times of the vowels in each stressed syllable. Children’s sensory processing of frequency, duration, intensity and AERT are therefore likely to be central to their ability to differentiate syllable stress patterns and prosodic hierarchies.

Research into the frequency sensitivity of children with DLD has produced mixed results, with some cohorts of language-impaired children being found to have reduced frequency discrimination skills [22,24], whilst other groups have not differed from age-matched controls [21,25]. Duration discrimination has reliably been found to be poorer in children with DLD [21,22,26], whilst tests of intensity discrimination have found no difference between children with DLD and age-matched controls [24,26]. Several studies have shown impaired discrimination of AERT in children with DLD [21,22,26,27], leading to our first investigations into impaired speech rhythm in DLD [26]. Children with DLD also have difficulties with non-linguistic aspects of rhythmic processing. For example, Corriveau and Goswami [28] asked children with DLD to tap to a metronome beat and found that they were considerably poorer at synchronising their taps with the metronome than either age-matched or language-matched control children at rates of 2 Hz and 1.5 Hz (rates that broadly correspond to typical inter-stress intervals found in speech, [29]). A widely-studied family, known as the KE family, some members of whom display a hereditary form of DLD characterised by articulation difficulties, have also been tested with tasks measuring sensitivity to non-speech pitch and rhythm. Affected members performed more poorly on tests of rhythmic perception and production, indicating a level of rhythmic difficulty for those who also displayed language difficulties [30]. Indeed, tapping to a beat is also impaired in children who stutter [31].

Regarding relations with linguistic processing, in their study of children with DLD, Cumming et al. [32] reported that individual differences in a speech rhythm matching task and a musical beat perception task were significant predictors of children’s scores in standardised measures of receptive and expressive language development. Those children with DLD who had better rhythm matching or better musical beat perception had better language scores than those with poorer rhythmic skills. Both Corriveau and Goswami [28] and Cumming et al. [32] reported that individual differences in beat synchronisation contributed unique variance to measures of language and literacy. Weinert [33] also linked rhythmic processing with language ability, finding that children with DLD who did more poorly in a rhythm discrimination task were also poorer at learning an artificial language. Finally, links between rhythmic processing and language skills have also been reported for typically developing children. Gordon et al. [34] found that performance in a test of rhythm discrimination correlated significantly with scores in expressive morpho-syntax in 6-year-old children with no language impairments, accounting for 48% of variance in scores. Accordingly, proficiency in rhythmic processing may be linked to better syntactic skills across the ability range.

One plausible reason for a relationship between rhythm and syntax could be that children with better rhythmic skills may be better at exploiting prosodic phrasing in order to bootstrap language learning [21,22,32]. There is evidence that prosodic phrasing contains cues to syntactic structure, and that both adult language-listeners and infant language-learners make use of these cues in order to parse the speech stream and comprehend language. For example, Price, Ostendorf, Shattuck-Hufnagel and Fong [35] found that adult listeners were able to disambiguate between two possible syntactic parsings of phonologically identical sentences by using prosodic features, such as intonational phrase boundaries and size of prosodic breaks (duration of pauses). Infant experiments have employed preference paradigms, in which infants aged between 7 and 10 months are played recordings with pauses inserted either clause-finally (i.e., coinciding with a syntactic boundary) or mid-clause [18]. The infants demonstrated a preference for stimuli where the pauses were clause-final. A similar preference was also demonstrated by 9-month-old infants for phrase-final pauses [19]. This indicates that, before the end of their first year, infants are already sensitive to the typical coincidence of prosodic and syntactic cues found in the language environment. Jusczyk et al. describe this use of prosodic cues as a ‘perceptual precategorisation’ [19] (p. 287), thought to enable a more detailed analysis of each resulting perceptual grouping. By aligning the segmentation of perceptual groups with meaningful grammatical units, this precategorisation process would serve perceptually to delimit alternatives, effectively chunking the continuous incoming speech stream and consequently enabling a more nuanced grammatical analysis to take place. By this means, efficient processing of the prosodic structure of speech could pave the way for efficient learning of syntactic organisation.

Whilst much research has been conducted on the nature of the grammatical deficit in DLD, little attention has been paid to the role that prosodic factors may play in the development of grammatical competence, and hence to the role that a difficulty with processing prosodic phrasing might have in the trajectory of the disorder. However, the infant work outlined above indicates that prosodic processing of rhythm patterns may lay the foundations for the discovery of grammatical units at an early stage of language development. In line with this perspective, Demuth has demonstrated that young typically developing children will vary their production of grammatical morphemes depending on the prosodic context. Accordingly, she has argued for a ‘Prosodic Licensing’ approach to syntactic development, in which the prosodic structure of a given language and the location of a particular grammatical morpheme in the prosodic contexts afforded by that language will interact to ‘license’ the use of particular morphemes by the young child [36,37]. Demuth and Tomas [37] argued that an understanding of how prosodic phonology operated to support morphological development in typical development could help to illuminate morpho-syntactic errors by children with DLD. Given our perceptual studies showing that children with DLD have difficulties in processing both speech and non-speech rhythm [21,22,32], children with DLD may also have difficulties in processing the rhythmic aspects of speech that can facilitate the overall acquisition of grammatical structure. If so, this could provide an acoustic, stimulus-driven account of the grammatical difficulties that typify the receptive and expressive language of children with DLD.

The current investigation explores children’s sensitivity to prosodic phrasing as a cue to the parsing of the speech stream into smaller, more manageable, grammatical units. Whilst prosodic and syntactic structures do not always coincide in natural speech, there is nonetheless a core area of children’s typical language exposure in which the two levels are tightly integrated, namely the realm of children’s oral and textual culture. Children’s stories and nursery routines draw heavily on rhythmic devices to structure language, as aspects of children’s linguistic life, such as nursery rhymes and clapping games, depend on the integration of repetitive language and repetitive rhythm. A further aspect of a typical child’s linguistic environment is children’s literature, which frequently relies heavily upon rhythm and rhyme. Many successful children’s authors build upon the playfulness of oral rhymes, with writing characterised by strong, repetitive rhyme and rhythm frameworks. We hypothesised that the predominance of rhythm and rhyme in these texts may serve a scaffolding function in developing children’s awareness of prosodic-syntactic units. Accordingly, we selected a representative story by former UK children’s laureate Julia Donaldson called ‘*Room on the Broom*’: a story with a strong rhythmic format [38].

The rhythmic format of *Room on the Broom* creates a tight integration of prosody and syntax and hence contains rich structural cues to grammar. The child is exposed to cues at multiple hierarchical layers, drawing their attention simultaneously to the phonological, prosodic and syntactic structure of the language. The property of rhyme emphasises the phonological structure of words by drawing attention to the onset-rime division, whilst also providing a guide to linguistic structure since each rhyme occurs at the end of a syntactic unit (be that clause or phrase). The overarching metrical structure also draws attention to the rhyme boundary point, since it occurs at regular intervals every four metrical feet. Within that metrical structure, there are further subdivisions into pairs of metrical feet, each of which also generally represents a complete syntactic unit. The metrical structure is therefore not an arbitrary form superimposed on the syntax of the text, but the two structures form a rich and highly integrated input which serves to highlight and reinforce the rhythmic and syntactic properties of language.

**Figure 1:**
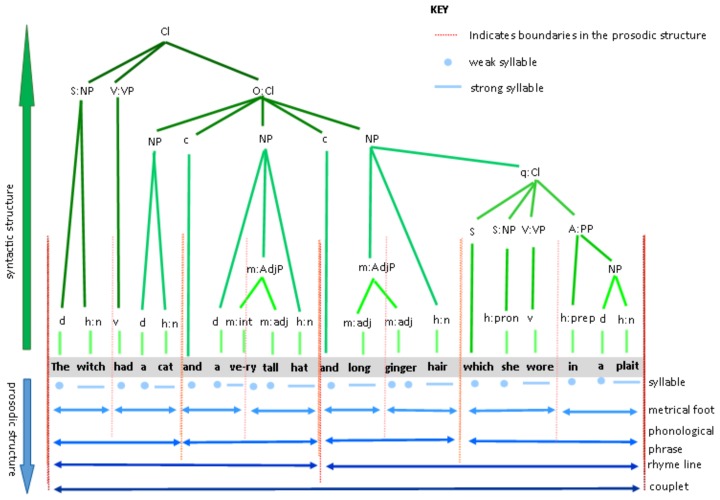
A diagram to illustrate the syntactic and prosodic structure of a line from Room on the Broom. Abbreviations: d-determiner; h:n-noun, head of noun phrase; v-verb; m:int-modifier:intensifier; m:adj-modifier:adjective; h:pron-pronoun, head of noun phrase; h:prep-preposition, head of prepositional phrase; c-conjunction, q-qualifier; Cl-clause; S-subject; O-object; A-adverb; NP-noun phrase; VP-verb phrase; PP-prepositional phrase.

An illustration is provided as Figure 1, which decomposes the structural embedding in the opening sentence of this popular children’s book. The figure marks out the major syntactic structures (shown above the text in green) and the major prosodic structures (shown below the text in blue). The figure shows that the major groupings in the syntactic structure are mirrored by major prosodic boundaries (the dashed red lines) in the prosodic structure. The prosodic boundaries are hierarchically nested such that the larger the prosodic-syntactic unit, the greater the overlap of boundary cues. Accordingly, the end of each rhyme line represents the combined boundary of four different levels of metrical analysis, as well as the boundary of a major syntactic unit. The prosodic structure is built around the stressed syllables, which serve to demarcate the end of a metrical foot (predominantly anapaest; i.e., weak-weak-Strong (wwS)). The symmetry is not faultless, as can be seen from ‘a very tall hat’, in which the lexical word ‘very’ crosses the boundary of the metrical foot; however, for the majority of the couplet, there is a strong coincidence of prosodic and syntactic boundaries. Given this level of dovetailing between the prosodic and syntactic structures, our study aimed to measure to what extent the children with DLD were able to integrate these two systems of representation.

## 2. Materials and Methods

### 2.1. Participants

Fifty-nine (59) children took part in the study. Children were divided into three groups: 13 had developmental language disorder (DLD group) Mean (*M*) age 102 months, range 77–140; 24 were age-matched typically developing controls (AMC group) *M* 107 months, range 77–132; and 22 were younger, language-matched controls (YLC group) *M* 66 months, range 57–74. All of the children were attending mainstream schools across state and private sectors in the East of England.

Children with DLD were recruited via their schools by asking teachers to nominate pupils whom they considered displayed difficulties with language. Those children identified by their teachers then completed four standardised language tests, the British Picture Vocabulary Scales-2nd Edition (BPVS II) [39], and three subtests of the Clinical Evaluation of Language Fundamentals UK-3rd Edition (CELF3^UK^) [40]: the Recalling Sentences, Concepts & Directions and Formulated Sentences subtests. Children who scored at or below −1.33 SD on at least two of the four tests went on to be included in the DLD group. Age-matched children (AMC group) were largely recruited from the same schools as the children with DLD and also completed the four standardised language tests. Only children scoring higher than −1 SD on all four tests were included in the study as part of the AMC group. The younger children (YLC group) all attended a single school who agreed to take part for this purpose. Children in the YLC group completed the BPVS II and the CELF3^UK^ Recalling Sentences subtest only. All children also completed the Block Design, Picture Completion and Digit Span subtests of the Wechsler Intelligence Scale for Children 3rd Edition (WISC III) [41] as measures of phonological memory and non-verbal intelligence quotient (IQ). Results of the standardised tests are displayed in Table 1.

As different groups did different tasks, one-way ANOVAs by group or independent samples t-tests were used to assess group differences. The matching was confirmed as the DLD group did not differ significantly from the AMC group on Age (months) (*p* = 0.675), whilst both the DLD and AMC groups were significantly older than the YLC group (*p* = 0.000). The DLD and YLC groups did not differ significantly from each other on measures of language (Recalling Sentences *p* = 0.434; BPVS II *p* = 0.641), whilst both groups were significantly different from the AMC group (*p* = 0.000). The DLD group were also significantly different from the AMC group on the additional language measures of Formulated Sentences and Concepts & Directions (*p* = 0.000).

For the IQ measures, the DLD group scored within one standard deviation of the standardised mean for both tasks, indicating that their non-verbal IQ was within typical norms; however, their scores as a group were nonetheless significantly lower than those of the AMC group (Picture Completion *p* = 0.017; Block Design *p* = 0.014). The DLD group also had a significantly lower Digit Span score than the AMC group (*p* = 0.000).

### 2.2. Materials

The aim of the experimental task was to investigate whether children were sensitive to the coincident boundaries of prosodic and syntactic units as exemplified in the rhythmic texts that form a central part of children’s literature. The rhyming couplets in the chosen text consisted of two lines, each of which contained four stressed syllables (in capitals):

*the WITCH had a CAT and a VEry tall HAT*

Each rhyme line was also composed of two syntactic units, each of which contained two stressed syllables (i.e., two metrical feet):

*the WITCH had a CAT and a VEry tall HAT*

This clear and regular correspondence between metrical and syntactic units continues throughout the text. From an analysis of the whole book, 10 couplets were chosen to form the stimulus set. Five couplets had the regular pattern:

__SwwS; wwSwwS e.g., ‘Down!’ cried the witch, and they flew to the ground,_wSwwS; wwSwwS They searched for the hat, but no hat could be found.

The other five couplets had the regular pattern:

wSwwS; wwSwwS e.g., Then out from a tree, with an ear-splitting shriekwSwwS; wwSwwS There flapped a green bird, with the bow in her beak.

To investigate whether metrical groupings influence detection of syntactic-prosodic units, three conditions were created: Metrical-Coincident; Metrical-NonCoincident; and NonMetrical-NonCoincident. A pause was created in the spoken recordings of the couplets to create the three different conditions, as detailed in Table 2.

It should be noted that the syntax in each version remains identical, only the prosodic grouping is altered. Accurate judgements therefore would not reflect syntactic knowledge per se, but rather intuitive knowledge of how prosody and syntax typically interact.

### 2.3. Recording

All stimuli were recorded in a soundproof booth by a female speaker of British English using a TASCAM DR-100 recorder via a SHURE SM58 condenser microphone. A regular beat was induced in the speaker using a priming metronome beat in one ear (not audible on the recording) with an inter-beat interval of 750 ms. The stimulus was then spoken so as to align the stressed syllables of the recording with the beats at 750 ms intervals. The precision of this timing was then verified and adjusted as necessary with Audacity software. The inserted pause was equivalent to the insertion of one silent stressed syllable interval, such that there was 1500 ms between the preceding and the following stressed syllable.

Each couplet was recorded in three different versions: Met-Co, Met-NonCo and NonMet-NonCo. The couplets were then arranged in three blocks of 10 couplets, with each block containing a counterbalanced mix of all three conditions (e.g., four Met-Co, four Met-NonCo and three NonMet-NonCo). Each couplet occurred only once in each block, and the order of couplets was fixed across blocks. Each block was listened to in a separate session, with the order of presentation of blocks across the three sessions randomised across participants. Each child ultimately listened to each block and therefore recorded scores for all three versions of each couplet.

### 2.4. Procedure

Each child completed the task individually in a quiet area at school. In the first testing session, the experimenter read the entire storybook to the child so that each child was familiar with the text as a whole. Each task block was then presented as part of a wider set of experimental tasks in subsequent sessions.

The task was contextualised by talking about how when reading out loud it was important to take a breath in a ‘sensible place, where it fits with the words’ because otherwise ‘it…sounds interrupted…like…this.’ It was then explained that they were going to hear someone reading the words from ‘Room on the Broom’ but that sometimes the reader would take a breath in a ‘funny place; where it sounds wrong; like it doesn’t fit’. The task was presented using a laptop computer running Presentation software with the children listening through Sennheiser HD650 headphones via a UGM96 soundcard. The corresponding picture from the book was displayed during the playback of each stimulus. Responses and Response Times were recorded using key presses on the laptop keyboard. Children were asked to press the key with the green ‘tick’ sticker if they thought the breath sounded like it was in a sensible place which fitted with the words, or the key with the pink ‘cross’ sticker if they thought it sounded wrong or interrupted. These buttons corresponded to the ‘L’ and ‘A’ buttons on the keyboard.

Each presentation of a block of 10 trials was preceded by three practice trials, during which children were given feedback to ensure they understood the task. This was followed by presentation of the 10 experimental stimuli, during which children were given only generic encouragement.

### 2.5. Auditory Threshold Estimation Tasks

Children in the AMC and DLD groups also completed four auditory threshold (AT) estimation tasks designed to probe sensitivity to four key acoustic indicators of stress in speech: Amplitude Envelope Rise Time (AERT); Frequency; Duration and Intensity. These were presented via the laptop computer using the Dino software program.

The AT tasks all followed a similar format in which, for each trial, the child heard three tones and was asked to choose which tone was different from the other two. Presentation was always in an AXB format where the middle tone (X) was always the reference tone, one of A and B was also the reference tone whilst the other differed from the reference by a stipulated amount (see below). Children were shown a picture of three cartoon animals and were told that each animal would make a noise and jump at the same time. Their job was to choose the animal that made the different sound. Responses were through mouse click or by pointing. The program provided continuous feedback, with correct answers rewarded with a colourful icon and incorrect answers indicated by an auditory sigh. Each block was preceded by five practice trials during which children received live feedback and further explanation of the task. Tasks were presented in a fixed order of Frequency, Intensity, AERT, Duration.

Frequency: Stimuli consisted of 200 ms tones played at 80.95 dB. The minimum frequency was 250 Hz (reference tone) and the maximum was 279.92 Hz. Increments between tones were of 0.0513 semitones. Children were asked to choose the tone with the different, higher sound.

Intensity: Stimuli consisted of 200 ms tones at a frequency of 250 Hz. The minimum intensity was 61.472 dB and the maximum was 80.95 dB (reference tone). Intensity intervals between levels were of 0.5128 dB. Children were asked to choose the tone with the different, quieter sound.

AERT: Stimuli consisted of 800 ms tones played at 80.95 dB at a frequency of 531.25 Hz. The minimum rise time was a 15 ms slope (reference tone) and the maximum was a 300-ms slope. Fall-off was consistent at 50 ms. Increments to the slope between levels were of 7.0377 ms. Children were asked to choose the tone with the different, gentler beginning.

Duration: Stimuli consisted of tones played 80.95 dB at a frequency of 250 Hz. The minimum duration was 400 ms (reference tone) and maximum duration was 595 ms. Increments in duration between levels were of 5.1282 ms. Children were asked to choose the tone with the different, longer sound.

The Dino program uses a staircasing procedure in order to estimate the auditory threshold. Trials begin with the maximum difference between stimuli (i.e., levels 1 and 40) and initially use a two-up, one-down procedure. This means that two correct answers result in a narrowing of the difference between stimuli, whilst one incorrect answer results in a widening of the difference between stimuli. After four reversals, the procedure is three-up, one-down. Initially, stimuli pairings change by eight levels in each stepchange (e.g., moving from levels 1:40 to levels 1:32); after four reversals, this becomes progressively four-, two- and one-level stepchanges. The final threshold figure is taken as the mean level from the fourth reversal.

Ethical approval for the study was obtained from the Cambridge Psychology Research Ethics Committee reference PRE.2009.02.

## 3. Results

### 3.1. Accuracy

Children’s scores were summed across blocks and calculated according to number of responses correct (i.e., identifying stimuli in condition Met-Co as correct with a ‘tick’ press and those in conditions Met-NonCo/NonMet-NonCo as incorrect with a ‘cross’ press). The maximum score was therefore 30, with a maximum score of 10 for each condition.

Due to software errors, two children from each group unintentionally listened to the same block presentation twice. These children’s scores were removed from the summary analysis. From a boxplot of scores by group, one AMC child appeared as an outlier. This was confirmed by calculating this child’s z-score; this child was still an outlier and so these scores were also removed. Scores for each of the conditions by group are given in Table 3.

As will be recalled, the different conditions were mixed together during task presentation to the child; however, in order to judge whether the groups differed in sensitivity to the task, d’ was calculated for each group. Hits were defined as selecting the ‘tick response for target tick and the cross response for target cross’. The resulting mean group values were AMC d’ = 2.442, DLD d’ = 1.395, YLC d’ = 1.045. A one-way ANOVA (DV d’) revealed that the AMC group was significantly more sensitive than the DLD group (*p* = 0.033) and the YLC group (*p* = 0.001) (Games–Howell corrections). The sensitivity of the DLD and YLC groups did not differ from each other. Accordingly, the DLD children were less sensitive to prosodic-syntactic groupings than would be expected for their age, but were not less sensitive to these groupings than would be expected for their language attainment levels.

In order to compare the groups in terms of accuracy of performance, a 3 × 3 repeated-measures ANOVA (Group: AMC, DLD, YLC; Condition: Met-Co, Met-NonCo, NonMet-NonCo) was conducted. The ANOVA showed no significant main effect of Condition, *F*(1.193,58.473) = 2.004, *p* = 0.160 (Greenhouse–Geisser correction); however, there was a significant effect of Group, *F*(2,49) = 12.077, *p* = 0.000 and the Condition*Group interaction was also significant, *F*(4,98) = 4.465, *p* = 0.002. Pairwise comparisons (Bonferroni) indicated that the AMC group scored more highly than both the DLD group (*p* = 0.011) and the YLC group (*p* = 0.000), whilst there was no significant difference in score between the DLD and YLC groups. This is consistent with the d’ analysis.

The significant Group*Condition interaction was explored by running a series of one-way ANOVAs for each condition. The ANOVAs revealed no main effect of group for the Met-Co condition, but a significant group effect for the Met-NonCo, *F*(2,20.810) =14.243, *p* = 0.000 and for the NonMet-NonCo, *F*(2,20.556) = 14.435, *p* = 0.000 (Welch’s *F*) conditions. Post-hoc tests (Games–Howell) showed that the AMC group were more accurate than the DLD and YLC groups in both of these conditions (Met-NonCo *p* = 0.009, DLD, *p* = 0.001, YLC; NonMet-NonCo *p* = 0.044, DLD, *p* = 0.000 YLC). The DLD and YLC groups did not differ significantly from each other in either condition.

**Figure 2:**
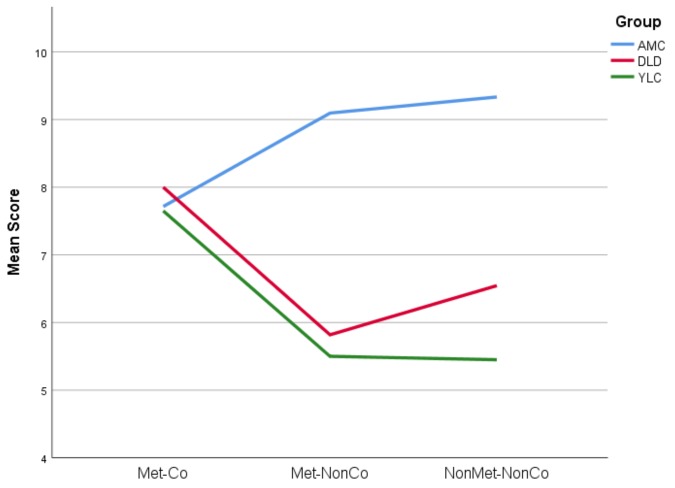
A graph showing the mean score for each condition by group. AMC: Aged-matched children; DLD: Developmental Language Disorder; YLC: younger, language-matched control.

Inspection of the graphed results (Figure 2) helps to illustrate the differing effect of condition for the three groups. As the graph shows, the AMC group scored more highly for the non-coincident Met-NonCo and NonMet-NonCo conditions than for the Met-Co condition, whilst the YLC group’s scores were lower for the non-coincident stimuli than the coincident Met-Co type. An unexpected pattern in the results was the relatively poor performance of the AMC group in the Met-Co condition. When compared to their performance in the non-coincident conditions, this suggests that the AMC children were slightly more likely to reject a correct rendition than to accept an incorrect one. The graph also indicates that the children with DLD were as accurate at identifying when the coincidence of prosodic-syntactic cues was correct (Met-Co) as were the AMC and YLC children. However, once these structures were disrupted, their performance fell markedly, suggesting poor sensitivity both to regular metrical groupings that did not coincide with a syntactic boundary (the Met-NonCo condition) and poor sensitivity to irregular groupings that did not coincide with a syntactic boundary. If the children with DLD were sensitive to metrical structure but did not relate this to syntactic structures, then we would expect lower accuracy for Met-NonCo than NonMet-NonCo. However, the performance of the DLD children in both disrupted conditions was statistically equivalent, suggesting that they were insensitive to both speech rhythm and its relationship to syntax.

### 3.2. Reaction Times

**Figure 3:**
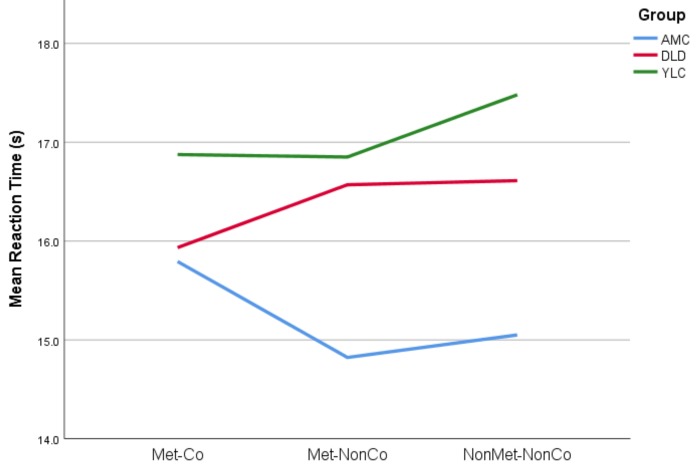
A graph of mean reaction times to each condition by group.

In order to explore group performance in more detail, reaction time data (RT) were also analysed. The mean RT was calculated for each child for each Condition (regardless of correctness of response). Data are shown in Figure 3 and Table 4.

A repeated-measures 3 × 3 ANOVA (Group: (AMC, DLD, YLC) × Condition: (Met-Co, Met-NonCo, NonMet-NonCo)) showed no significant effect of condition on Reaction Times, *F*(2,98) = 0.796, *p* = 0.454, nor of group, *F*(2,49) = 2.968, *p* = 0.061. However, there was a significant Group*Condition interaction, *F*(4,98) = 2.877, *p* = 0.027.

In order to explore the Group*Condition interaction, a series of one-way ANOVAs were run for each Condition. There was no significant effect of group for the Met-Co and the Met-NonCo conditions. There was, however, a significant effect of group for the NonMet-NonCo task, *F*(2,49) = 4.667, *p* = 0.014. Pairwise comparison (Games–Howell) of the means for each group showed a significant difference between the YLC and AMC groups in the NonMet-NonCo condition: the younger children were significantly slower (*p* = 0.012). Furthermore, inspection of the graph in Figure 3 shows that both the YLC and DLD groups tended to be slower in response than the AMC group. Accordingly, for the two non-coincident tasks, the DLD children appeared to respond within a similar timeframe to the younger YLC children.

For completeness, the group*condition interaction was also explored using one-way repeated-measures ANOVAs by group. There were significant effects of condition for the AMC children, *F*(1.339,26.774) = 4.181, *p* = 0.04 (Greenhouse–Geisser correction) but no significant effect of condition for the DLD children, *F*(2,20) = 1.286, *p* = 0.298 nor for the YLC group, *F*(2,38) = 1.687, *p* = 0.199. For the AMC group, despite the significant overall effect of condition, post-hoc pairwise comparisons (Bonferroni) showed no significant differences between conditions, although there was a trend for the responses in NonMet-NonCo to be quicker than those of the Met-Co (*p* = 0.077). In other words, there was a tendency for the AMC group to take longer to decide that the coincident stimulus was correct than to decide that the non-coincident stimulus was incorrect, even though they performed well in both conditions. This result tallies with observations during testing: AMC children often pressed the [x] button as soon as they heard the first non-coincident boundary, immediately confident that this presentation was ‘wrong’. In order to be sure that the coincident stimulus was correct, however, the stimulus had to be listened to in its entirety. This may explain this difference in response time trends for the AMC group.

A different effect was observed for the DLD group, who rarely pressed the response buttons before the full stimulus was played. This is reflected in the lack of variation in their response times. It seems that, for the DLD children, in marked contrast to the AMC children, there was no confident decision-making about aberrant prosodic-syntactic groupings reflected in quicker response times. The DLD children puzzled for an equally long time over the coincident stimuli as they did over the non-coincident stimuli. In doing so, they presented a response profile that was statistically comparable to the younger language-matched children.

### 3.3. Acoustic Threshold Estimation Tasks

The AMC and DLD groups both completed the four AT tasks in AERT, Frequency, Duration and Intensity. Two scores were not recorded by the software: one Frequency score (one DLD child) and one Intensity score (one AMC child). A series of independent samples t-tests was conducted to examine any differences in acoustic sensitivity between the two groups (see Table 5).

The AMC group had significantly lower thresholds (i.e., were able to discriminate more fine-grained differences between stimuli) than the DLD group for the conditions of AERT, Frequency and Duration, whilst there was no significant difference between groups for Intensity. The finding that the AMC and DLD groups performed the Intensity threshold task at equivalent levels shows that the attentional load of the task is not a factor in explaining group performance.

A correlation analysis between acoustic threshold and accuracy score on the experimental task revealed significant correlations between task performance and sensitivity to the acoustic features of AERT (*p* = 0.027), Duration (*p* = 0.004) and Frequency (*p* = 0.000), with the greater the sensitivity to acoustic differences, the more accurate the performance on the task (see Table 6).

The children with DLD therefore had larger thresholds (i.e., required a greater difference between stimuli in order to discriminate) for the acoustic measures of Frequency, Duration and AERT and all three of these measures were significantly correlated with success on the experimental task.

A series of three-step fixed order multiple regressions were carried out to explore the unique contributions of each of the acoustic parameters of Frequency, Duration and AERT to success on the task once age and non-verbal IQ (NVIQ) were controlled. NVIQ was taken as the mean score across the two subtests of Picture Completion and Block Design. Table 7 shows the results of the equations with accuracy as the dependent variable (i.e., the child’s overall score summed across all three conditions).

Age was not a significant predictor of performance in this task (*p* range = 0.165–0.303); however, NVIQ contributed significant amounts of unique variance for all three equations (range 29.3–35.1%, *p* range = 0.000–0.001). The greatest unique variance accounted for by the AT tasks was for Frequency (7.1%) followed by Duration (5.8%), although neither was significant once NVIQ was controlled (*p* = 0.071, 0.105, respectively). AERT also failed to make a significant contribution to overall accuracy once NVIQ was controlled (*p* = 0.374) contributing 1.8% of unique variance, the smallest amount. The significant correlations between sensitivity to AERT, Duration and Frequency and overall score may therefore have been partly mediated by NVIQ, with the acoustic cues of Frequency and Duration providing smaller (non-significant) additional contributions to the variance in score.

A further set of three-step fixed order regressions was calculated to explore the relationship between the different acoustic parameters and the overall mean response time (calculated by summing all RT values for each child and dividing by 30). These are shown in Table 8.

In these regressions, Age was a significant predictor of RT, explaining between 18.8% and 19.3% of unique variance. NVIQ did not contribute significantly to the model, and nor did any of the three acoustic parameters. This suggests that the speed of responding was primarily mediated by the age of the participants, even though the younger LMC children were not included in these analyses. The negative b values indicate that the older the participants were, the faster their responses.

## 4. Discussion

This study set out to investigate the influence of phrasal metrical hierarchies of acoustic stress patterns on children’s capacity to recognise appropriate prosodic-syntactic groupings. In children’s literature, the metrical regularities of the texts typically serve to emphasise the syntactic structures of language through the coincidence of prosodic and syntactic boundaries. Here, children listened to different readings of phrases from a children’s story, and were asked to indicate when the reader took a breath in a ‘funny place, where it sounds wrong, like it doesn’t fit’. Two types of disruption were tested, breaths that created metrical groupings that conflicted with syntactic groupings (Met-NonCo) and breaths that violated both metre and syntax (NonMet-NonCo). As children with DLD have known acoustic difficulties with stress, indexed by their insensitivity to amplitude envelope rise time (AERT), and acoustic difficulties with grouping, indexed by their insensitivity to duration [21,22], this may affect their ability to use the prosodic-syntactic grouping typical of representative texts in children’s literature to aid grammatical learning. If children have robust knowledge of how prosody and syntax interact, then any violation of these coinciding units should be readily identified. Alternatively, if children are able to detect metrical patterns but are unable to relate these to the overall prosodic-syntactic structure, then phrases in the condition in which there is an acoustic metrical rhythmic structure that does not coincide with the syntax (condition Met-NonCo) should prove more difficult to reject than phrases in the condition in which there is no consistent metrical acoustic pattern (condition NonMet-NonCo). If the children with DLD are insensitive to both acoustic prosody and its relationship with syntax, then there should be no difference in performance between the three different conditions.

Overall, the DLD children were indeed less sensitive to the prosodic-syntactic groupings that we used, as their d’ scores were significantly lower than that of the age-matched control (AMC) children. Nevertheless, the DLD children were as sensitive to the prosodic-syntactic groupings as the younger language-matched control (YLC) children, as their d’ scores were statistically equivalent. However, their reduced sensitivity did not reflect a lack of effort in the task. The slowed response times of the DLD and YLC children showed that they were analyzing the non-coincident phrases, as they were slower to respond in the two non-coincident conditions. The DLD group were impaired compared to the AMC group at detecting violations of prosodic-syntactic units, and this was shown by both their response time data and their accuracy data. The accuracy of the DLD group was comparable to that of the younger children. This is the pattern that we would expect if DLD children have difficulty in processing language metre and its relationship with syntactic structures. From the accuracy scores, it seems that the most significant impact of metrical grouping appears to be the attention that it draws to the prosodic-syntactic unit, rather than its temporal regularity per se. If metrical regularity alone (regardless of syntax) were influencing responses, we would expect a higher error rate in the Met-NonCo condition than NonMet-NonCo, with DLD children responding to the regular metre in the former condition and judging the stimulus phrases as acceptable. The data did not support this explanation, as the slight difference visible in Figure 2 was not significant.

Indeed, the response time data showed that the DLD and YLC children did not differ in their response times across conditions. If DLD children were confident in using prosody to detect syntactic boundaries regardless of metre, we would expect swift responses to these violations (fast responding for both Met-NonCo and NonMet-NonCo conditions, as found for AMC children). Alternatively, if the DLD children were able to detect the metrical grouping but could not readily relate that perceptual structure to the syntactic groupings, then we would expect Met-NonCo stimuli to produce slower responses due to the conflicting information. This was not the case, suggesting that the DLD children could neither detect the metrical grouping nor integrate it with their expectations of prosodic-syntactic groupings. Inspection of the response time data showed that the children with DLD did not make early detections of errors, almost always choosing to listen to the whole recording. Accordingly, the children with DLD were unable to systematically determine whether structural boundaries had been violated. Their performance in both accuracy and speed of response resembled that of the younger children (YLC group), suggesting a developmental delay in their ability to integrate prosodic and syntactic structures.

Overall, the data suggest that younger children and DLD children have less well-developed schema for how prosody and syntax interact compared to older typically developing children and therefore that this is an aspect of language processing that continues to develop throughout childhood. The DLD children appear to have underdeveloped schema for the interaction of prosody and syntax for their age. This suggests that they may not be processing all of the cues available to them in segmenting the speech stream into prosodic units and grammatical clauses. Instead, they responded similarly to the younger children. Note that the syntax itself was identical in all three conditions, so the test is not one of grammatical structures per se, but of how these structures interact with prosodic units in typical speech.

The range of responses here sits interestingly between experiments with infants, which have shown that infants are sensitive discriminators of pauses inserted within clauses or at clause boundaries [42], and those with adults, who also judge sentences where pauses coincide with phrasal boundaries to be more natural [19]. The older AMC children were more adult-like in their responses, being able to judge both the Met-Co sentences as being natural and the Met-NonCo and the NonMet-NonCo sentences as ‘sounding funny’. The question, however, is why the DLD and, particularly, the YLC children have relatively poor accuracy for the non-coincident stimuli if 9-month-old infants are sensitive to these boundaries. One explanation could lie in task demands. In our experiment, the children were asked to decide explicitly which was the ‘correct’ version, and so this required a greater degree of metalinguistic awareness than the infants in Jusczyk et al.’s passive listening study [19]. This raises an important conceptual difference between ‘sensitivity’ in the sense of discriminating between prosodic structures and ‘awareness’ in the sense of consciously noticing the significance of any discriminated difference. It is possible that the children with DLD were sensitive to the differences between the prosodic structures in the stimuli but were unable to determine whether this observed difference resulted in a pragmatic difference: that the breaks were in a ‘funny place’. As we also collected acoustic data, however, and found reduced sensitivity for DLD children in three of the four acoustic parameters that contribute to stress perception, this seems unlikely. It seems more likely that the children’s lack of pragmatic awareness stemmed from their poorer discrimination of the acoustic parameters that enabled reliable identification of the prosodic structures. This perceptual difficulty then reduced the ability of the DLD children to integrate information about perceptual prominence with syntactic expectations.

On the basis of our previous acoustic work [21,22,26,28], we proposed that children with DLD may be delayed in developing schema for prosodic-syntactic hierarchies because of impaired sensory processing. In English, the four acoustic parameters that we measured of AERT, duration, frequency and intensity combine to give the percept of stress and thereby linguistic rhythm [23]. Accordingly, acoustic sensitivity to these parameters is likely to influence linguistic rhythm development. The DLD group had significantly higher thresholds for AERT, duration and frequency than did the AMC group, whilst they did not differ in intensity thresholds. This pattern of responses accords with previous studies that have found that children with DLD have impairments in discriminating AERT [21,26,27,43] and duration [21,22,26]. Some studies have also found a frequency impairment [22,24], but see also [21,25]. Poorer sensitivity in these acoustic tasks has previously been associated with poorer performance on tasks probing linguistic stress [21]. Here, we also found a significant association between the acoustic thresholds for AERT, duration and frequency and the capacity to detect violations of prosodic-syntactic boundaries. This suggests that the less sensitive auditory systems of children with DLD may be impacting upon their perception and subsequent integration of prosodic cues with larger-grained syntactic units. However, once non-verbal IQ was controlled in a series of regression equations, none of the acoustic parameters measured accounted for unique variance in task performance. This contrasts with some of our earlier DLD studies, where both AERT and duration measures have explained unique variance in language tasks even after NVIQ has been controlled [22,26]. Processing prosody across larger phrasal units requires the tracking of relative acoustic hierarchies across time and then integrating this lower-level phrasing into the overall acoustic hierarchy. If children with DLD need greater acoustic differentiation between phrases in order to discriminate the overall acoustic hierarchy, then the less salient stress cues available in natural language may result in demarcations in the signal being missed. This may in turn lead to a failure to establish schema (relative stress templates) for the hierarchical relationships necessary for interpreting prosodic structure at a phrasal and clausal level.

The coincidence of the acoustic cues that create the prosodic-syntactic structure is particularly salient in repetitive and rhythmic children’s literature, which was used to generate the stimuli used in this experiment. In stories such as *Room on the Broom*, there is reciprocal cuing of prosodic and syntactic elements such that sensitivity to one facet of the structure should facilitate processing of the other facet. However, our results suggest that this was not the case for the DLD children. Unlike typically developing children of the same age, they were unable to detect the prosodic-syntactic mismatches, suggesting that they are not yet proficient in integrating the two structural systems. Instead, they performed like the younger children in the experiment. This suggests a developmental trajectory for the development of prosodic-syntactic schemata in which the children with DLD exhibit delayed development. If children with DLD are in general slower to establish prosodic hierarchies, possibly due to poorer acoustic sensitivity, then it could be that they require greater exposure to structured linguistic input than do typically developing children to attain a similar developmental level.

It has previously been found that infant-directed speech (IDS) is much richer in acoustic cues to linguistic features than adult-directed speech (ADS) and that IDS can facilitate syntactic boundary detection in infants when compared with ADS [19,42]. Studies of the acoustic characteristics of IDS have found that the rhythmic focus rapidly shifts as the infant ages [44]. It could be therefore that for children with DLD, a longer period of structured prosodic input is required if sensitivity to the meaning of the units is to develop. If children with DLD are less efficient at discovering these acoustic cues to syntactic boundaries, and as IDS changes rapidly with the age of the child, it could be that children with DLD end up ‘missing out’ on this crucial early aspect of language acquisition: the incoming signal ‘moves on’ before their system is ready to cope with a less-structured and salient input. Such a scenario would have significant implications for language development. Morgan and Saffran [45] argued that prosody should be regarded as a kind of parameter-setting device, providing a rough categorisation of the input into smaller units and thereby constraining the amount of input, which is then subject to further analysis, for example by statistical learning. If this is the case, then sensitivity to prosodic units would be a powerful tool in the process of discovering grammatical units. In this view of language acquisition, poorer sensitivity (in terms of acoustic, stimulus-driven sensitivity) to metrical hierarchies of stress patterns would mean that constraining parameters fail to be set in chunking the input stream. Accordingly, a subsequent analysis would be carried out across much greater chunks of input, resulting in a far more unwieldy task. This in turn would lead to difficulty in segmenting language into grammatical units, such as clauses and phrases, with knock-on implications for acquiring smaller-grained aspects of morphology: exactly the kinds of linguistic difficulties that characterise children with DLD.

## 5. Conclusions

In conclusion, our results suggest that children with DLD have poorer sensitivity to the acoustic cues to linguistic rhythm that enable the creation of prosodic-syntactic schemata. This difficulty in recovering metrical hierarchies of acoustic stress patterns impairs their ability to capitalise on the prosodic cues to syntax present in speech which bootstrap grammatical competence. If prosodic cues enable more efficient parsing of the speech stream, then explicitly teaching children to listen for these acoustic stress cues may increase their ability to integrate prosody and syntax. Via such instruction, children’s capacity to derive grammatical structure from prosodically driven input could be increased. Accordingly, interventions using rhythmic children’s texts to highlight this congruence of prosody and syntax could theoretically be of great value in scaffolding grammatical development in children with DLD.

## Figures and Tables

**Table 1:** Results of standardized tests by group (Language: raw scores; intelligence quotient (IQ): scaled scores): one-way ANOVAs and independent samples *t*-tests.

Test	AMC Mean (SD)	DLD Mean (SD)	YLC Mean (SD)	*df*	*F*	*p*
Age (months) ^b,c^	107.33 (16.447)	102.31 (17.504)	66.18 (4.532)	2, 23.104 ^d^	89.068	0.000
**Language**						
Recalling Sentences ^a,b^	46.38 (12.917)	21.15 (6.950)	24.29 (7.309)	2, 33.565 ^d^	32.388	0.000
BPVSII ^a,b^	98.92 (21.279)	71.54 (11.163)	67.73 (13.364)	2, 34.879 ^d^	18.686	0.000
				***df***	***t***	***p***
Formulated Sentences ^a,^	31.58 (7.638)	16.08 (5.499)	-	35	6.452	0.000
Concepts & Directions ^a,^	25.00 (4.283)	14.54 (6.293)	-	18.181 ^d^	5.359	0.000
**IQ ^e^**						
Picture Completion ^a,b^	10.42 (2.518)	8.46 (1.664)	-	35	2.510	0.017
Block Design ^a,b^	10.42 (2.888)	8.15 (1.725)	-	35	2.577	0.14
Digit Span ^a,b^	9.96 (1.574)	6.62 (1.193)	-	35	6.675	0.000

^a^ Aged-matched children (AMC) > Developmental Language Disorder (DLD); ^b^ AMC > younger, language-matched control (YLC); ^c^ DLD > YLC; ^d^ adjusted *F* and *df* used due to significant Levene’s test; ^e^ IQ subtests are scaled scores: *M* = 10, SD = 3. BPVSII, British Picture Vocabulary Scales-2nd Edition.

**Table 2:** Condition Characteristics with example stimuli.

Condition	Description	Example Couplet	Metrical Structure	
**Metrical: Coincident** **(Met-Co)**	A pause at the end of every two metrical feet. This pause therefore coincided with natural prosodic and syntactic groupings.	‘Down!’ cried the witch /	__S	wwS
		and they flew to the ground /	wwS	wwS
		they searched for the hat /	_wS	wwS
		but no hat could be found	wwS	wwS
**Metrical:** **NonCoincident** **(Met-NonCo)**	A pause that created regular metrical groupings but that did not coincide with syntactic boundaries.	‘Down!’ cried /	__S	w
		the witch and they flew to /	wSw	wSw
		the ground they searched for /	wS_	wSw
		the hat but no hat could /	wSw	wSw
		be found	wS	
**Non-Metrical: Non-Coincident** **(NonMet-NonCo)**	A pause that created irregular rhythmic groupings and also did not coincide with syntactic boundaries	‘Down!’ cried the witch and they flew/	__S	wwSwwS
		to the ground they searched /	wwS	wS
		for the hat but no /	wwS	ww
		hat could be /	Sww	
		found	S	

**Table 3:** Accuracy scores by condition and group.

Score	AMC Mean (SD)	DLD Mean (SD)	YLC Mean (SD)
Overall Score	26.14 (3.692)	20.36 (5.519)	18.60 (5.995)
Met-Co	7.71 (3.002)	8.00 (2.449)	7.65 (2.183)
Met-NonCo	9.1 (1.261)	5.82 (2.857)	5.5 (3.456)
NonMet-NonCo	9.33 (1.278)	6.55 (3.236)	5.45 (3.268)

**Table 4:** Reaction Times (s) by Condition and Group.

	Met-CoMean (SD)	Met-NonCoMean (SD)	NonMet-NonCoMean (SD)
**AMC**	15.7932 (1.4338)	14.8225 (3.0666)	15.0502 (2.2551)
**DLD**	15.9342 (2.4686)	16.5701 (3.3560)	16.6115 (2.6472)
**YLC**	16.8754 (2.5224)	16.8506 (2.9466)	17.4805 (2.8310)

**Table 5:** Results of *t*-tests by group for auditory threshold (AT) tasks.

Task	AMC Mean (SD)	DLD Mean (SD)	*df*	*t*	*p*
**AERT (ms) ^a^**	114.628 (76.0437)	207.193 (69.971)	30	−3.357	0.002
**Frequency (Semitones) ^a^**	0.5681 (0.4428)	1.3747 (0.5117)	29	−4.512	0.000
**Duration (ms) ^a^**	79.8949 (39.1790)	138.4288 (47.8637)	30	−3.720	0.001
**Intensity (dB)**	−2.9863 (1.51905)	−3.291244 (1.5838)	29	0.527	0.602

^a^ AMC < DLD. AERT, Amplitude Envelope Rise Time.

**Table 6:** Correlation coefficients (Pearson one-tailed) for AT tasks and accuracy score.

Task	Duration	Frequency	Intensity	Task Score
AERT	0.458 **	0.794 ***	−0.123	−0.343 *
Duration		0.530 **	−0.084	−0.524 **
Frequency			−0.107	−0.553 **
Intensity				−0.077

* *p* <0.05; ** *p* <0.01; *** *p* <0.001.

**Table 7:** Stepwise regressions showing the unique variance in children’s accuracy in judging metrical and syntactic boundaries contributed by the different auditory processing measures.

	b	SEb	β	*t*	*p*	Δ*R*^2^	*p*
**Model 1**							
Age	−0.011	0.047	−0.036	−0.237	0.814	0.037	0.303
NVIQ	1.030	0.378	0.468	2.722	0.011	0.351	0.000
Frequency	−2.431	1.293	−0.314	−1.880	0.071	0.071	0.071
**Model 2**							
Age	0.008	0.054	0.022	0.140	0.889	0.063	0.165
NVIQ	1.076	0.433	0.435	2.482	0.019	0.293	0.001
Duration	−0.029	0.018	−0.288	−1.673	0.105	0.058	0.105
**Model 3**							
Age	0.014	0.056	0.041	0.255	0.801	0.063	0.165
NVIQ	1.309	0.413	0.529	3.168	0.004	0.293	0.001
AERT	−0.009	0.010	−0.145	−0.903	0.374	0.018	0.374

Note: b = unstandardised beta; SEb = standard error of b; β = standardized beta; ΔR^2^ = change in R^2.^

**Table 8:** Stepwise regressions showing the unique variance in children’s response time in judging metrical and syntactic boundaries contributed by the different auditory processing measures.

	b	SEb	β	*t*	*p*	Δ*R*^2^	*p*
**Model 1**							
Age	−0.059	0.029	−0.360	−2.016	0.054	0.188	0.015
NVIQ	−0.291	0.234	−0.254	−1.243	0.225	0.048	0.193
Frequency	−0.166	0.801	−0.041	−0.207	0.837	0.001	0.837
**Model 2**							
Age	−0.059	0.028	−0.367	−2.083	0.047	0.193	0.012
NVIQ	−0.301	0.228	−0.263	−1.321	0.197	0.048	0.186
Duration	−0.003	0.009	−0.062	−0.316	0.754	0.003	0.754
**Model 3**							
Age	−0.058	0.028	−0.360	−2.046	0.050	0.193	0.012
NVIQ	−0.269	0.211	−0.235	−1.275	0.213	0.048	0.186
AERT	0.000	0.005	−0.005	−0.029	0.977	0.000	0.977
